# On the Use of Injections of Rhatany in the Treatment of Fissure of the Anus, Prolapsus Ani, Leucorrhœa, &c.

**Published:** 1842-04-23

**Authors:** 


### THE MEDICAL EXAMINER.

PHILADELPHIA, APRIL 23, 1842.

*Rhatany in Fissure of the Anus.*—The N. Y. Medical Gazette, April 13, says: "We find in the report of the Clinique of Prof. Trousseau, at Necker Hospital, four cases in which this very troublesome and painful affection has been cured by simple enemata of extract of Rhatany. The professor remarks that similar cases have been published before; they escaped our notice, however; the remedy is new to us and we shall most assuredly make trial of it the first opportunity that offers. It is indeed a most important improvement if we can substitute for a surgical operation, which is always painful and sometimes even dangerous, (for M. Trousseau reminds us that a distinguished Parisian Professor died from the operation,) a remedy so simple, so easily administered, and so perfectly innoxious as the injection of Rhatany root."

We have used the injections of rhatany in cases of fissure of the anus, prolapsus ani, leucorrhœa, menorrhagia, with great advantage. For the employment of this remedy in fissure of the anus, we are indebted to M. Bretonneau, of Tours, (see Med. Examiner for 1840, p. 551.) In our last volume (for 1841, p. 292,) we published some cases by Drs. Johnston and Biddle, in which the rhatany had been successfully used in the treatment of fissure and prolapsus of the anus, and leucorrhœa. We have since employed it in cases of the same character, and with the best results.

